# Comparison of modulation efficiency between normal and degenerated primate retina

**DOI:** 10.3389/fcell.2024.1419007

**Published:** 2024-07-31

**Authors:** Yongseok Yoo, Seongkwang Cha, Yong Sook Goo

**Affiliations:** ^1^ School of Computer Science and Engineering, Soongsil University, Seoul, Republic of Korea; ^2^ Department of Physiology, College of Medicine, Chungbuk National University, Cheongju, Republic of Korea; ^3^ Biomedical Research Institute, Chungbuk National University Hospital, Cheongju, Republic of Korea

**Keywords:** retinal prosthesis, retinal ganglion cell, electrical stimulation, spontaneous firing, inter-spike interval, modulation efficiency

## Abstract

With electrical stimulation, retinal prostheses bypass dysfunctional photoreceptors and activate the surviving bipolar or retinal ganglion cells (RGCs). Therefore, the effective modulation of RGCs is crucial for developing retinal prostheses. Substantial research has been performed on the ability of an electrical stimulus to generate a reliable RGC response. However, different experimental conditions show varying levels of how well the electrical stimulation evokes RGC spikes. Therefore, in this study, we attempted to extract an indicator to understand how the electrical stimulation effectively evokes RGC spikes. Six cynomolgus monkeys were used: three as controls and three as an N-methyl-N-nitrosourea (MNU)-induced retinal degeneration model. The retinal recordings were performed using 8 × 8 multi-electrode arrays (MEAs). Electrical stimulation consisted of symmetrical biphasic pulses of varying amplitudes and durations. The number of stimulation conditions that resulted in significantly higher post-stimulation firing rates than pre-stimulus firing rates was defined as the modulation efficiency ratio (MER). The MER was significantly lower in degenerated retinas than in normal retinas. We investigated the relationship between the variables and the MER in normal and degenerated primate RGCs. External variables, such as duration and inter-electrode distance, and internal variables, such as average firing rates and statistics (mean, standard deviation, and coefficient of variation [CV]) of inter-spike intervals (ISIs) of spontaneous spikes, were used. External variables had similar effects on MER in normal and degenerated RGCs. In contrast, internal variables affected MER differently in normal and degenerated RGCs. While in normal RGCs, they were not related to MER, in degenerated RGCs, the mean ISIs were positively correlated with MER, and the CV of ISIs was negatively correlated with MER. The most important variable affecting MER was the mean ISI. A shorter ISI indicates hyperactive firing in the degenerated retina, which prevents electrical stimulation from evoking more RGCs. We believe that this hyperactivity in degenerated retinas results in a lower MER than that in the normal retina. Our findings can be used to optimize the selection of stimulation channels for *in vitro* MEA experiments and practical calibration methods to achieve higher efficiency when testing retinal prostheses.

## 1 Introduction

Retinal prostheses have been the subject of extensive research (Humayun et al., 2012; Lorach et al., 2015; Stingl et al., 2015; Fujikado et al., 2016; Weiland et al., 2016; Ayton et al., 2020). These medical devices replace the function of dead or dysfunctional photoreceptors. An epiretinal prosthesis can function by applying direct electrical stimulation to the surviving bipolar cells (BCs) or retinal ganglion cells (RGCs), whereas subretinal prostheses utilize incident light to trigger electrical stimulation via photodiodes (Lorach et al., 2015; Stingl et al., 2015). Ongoing research and development efforts aim to improve the efficacy, resolution, safety, and overall impact on the quality of life of individuals with visual impairments.

The development of retinal prostheses requires knowledge of how retinal neurons respond to electrical stimulation. The bipolar and ganglion cells are the main targets of electrical stimulation. The effect of electrical stimuli on the retina is most often determined by recording the responses of ganglion cells *in vitro* (Jensen et al., 2003; Jensen et al., 2005; Fried et al., 2006; Jensen and Rizzo, 2008; Sekirnjak et al., 2008; Jensen and Rizzo, 2009; Goo et al., 2011a; Jepson et al., 2013; Ahn et al., 2015; Weitz et al., 2015; Ahn et al., 2022; Ahn et al., 2023; Cha et al., 2023; Gogliettino et al., 2023). Consequently, electrical stimuli are classified according to their mechanism of action on RGCs into direct and indirect (network-mediated) stimulations. Direct stimulation of RGCs refers to a stimulus that depolarizes the ganglion cell membrane, causing RGCs to fire a spike without presynaptic input. Direct stimulation of RGCs causes them to fire a spike within a millisecond of the stimulus presentation (Sekirnjak et al., 2006; Jepson et al., 2013). Although direct stimulation can control RGC activity with fine temporal precision, axonal stimulation often makes it difficult to achieve a high spatial resolution (Weitz et al., 2013; Weitz et al., 2015). The indirect stimulation of RGCs is achieved by stimulating BCs, which in turn activate postsynaptic ganglion cells. The rationale for indirect stimulation is that the remaining retinal circuitry processes and refines the stimulus to produce a more natural ganglion cell output that mimics the physiological response. Therefore, our group has focused on the indirect stimulation of RGCs (Ahn et al., 2022; Cha et al., 2022; Ahn et al., 2023; Cha et al., 2023).

Substantial research has been performed on the ability of an electrical stimulus to generate a reliable RGC response in terms of the electrode location and the stimulus parameters, such as stimulus amplitude, duration, frequency, and pulse shape.

Epiretinal cathodic stimulation of ganglion cells has lower thresholds than anodic stimulation (Jensen et al., 2005; Ahn et al., 2015). Subretinal anodic stimulation has lower ganglion cell thresholds than cathodic stimulation has (Boinagrov et al., 2014). RGCs are most responsive to fast voltage changes to 1,000 Hz stimulation, while low-frequency (5–25 Hz) sinusoids can activate BCs, avoiding the activation of underlying ganglion cell axons (Freeman et al., 2010; Grosberg et al., 2017). BCs are known to be more responsive to 100 Hz stimulation, whereas photoreceptors are more responsive to 10 Hz stimulation (Freeman et al., 2010; Twyford and Fried, 2016). From the strength-duration curve, the rheobase current and chronaxie can be derived. For ganglion cells, chronaxie, which is the stimulus pulse duration found at twice the rheobase current, has been measured to be between 0.08 and 0.6 ms (Jensen et al., 2005; Sekirnjak et al., 2006; Ahuja et al., 2008; Eickenscheidt et al., 2012; Weiland et al., 2016).

Previous research greatly improves our understanding of how to generate reliable RGC responses with electrical stimulation. However, all the information is constrained by the experimental conditions, such as animal species, electrode configuration, electrical stimulation pulse, and so on. Sekirnjak et al. (2006) thoroughly reviewed all the preceding *in vitro* and *in vivo* experiments regarding animal species, electrodes, stimulation pulses, and threshold values. However, the comparison of different experiments makes it difficult to demonstrate how well electrical stimulation evokes RGC spikes. Therefore, in this study, we attempted to extract an indicator of modulation efficiency of an RGC, to quickly understand how electrical stimulation effectively evokes RGC spikes.

Specifically, we examined the effects of external and internal variables on the modulation efficiency in activating normal and degenerated primate RGCs using a 64-channel multi-electrode array (MEA). External variables include how the electrical current is delivered to an RGC, such as the charge of the electrical stimulation and the physical distance between the stimulating and recording electrodes. For internal variables, we focused on the intrinsic properties of an RGC that can be measured extracellularly. Spontaneous spiking patterns prior to electrical stimulation were summarized as internal variables.

Electrical stimulation studies with normal primate retinas related to retinal prostheses have been performed because the non-human primate (NHP) retina is the best animal model to mimic the human retina (Sekirnjak et al., 2008; Jepson et al., 2013; Gogliettino et al., 2023). However, to the best of our knowledge, there are no reports except our previous publication (Cha et al., 2023) that compared normal and degenerated NHP RGC related to retinal prostheses. The use of a drug-induced primate degeneration model in this study provided a unique opportunity to explore the differences between normal and degenerated RGCs in terms of modulation efficiency. The controlled environment of the *in vitro* setting allowed for a detailed analysis of how various factors influence the excitability and modulation of RGCs, providing insights that may contribute to the development of more effective retinal prostheses and treatments for vision restoration.

## 2 Materials and methods

### 2.1 Animals

Retinas were obtained from six adult male Cynomolgus monkeys (*Macaca fascicularis*). Retinal degeneration was induced in three monkeys by injecting them with N-methyl-N-nitrosourea (MNU) 14–21 weeks before sacrifice. We have previously reported the detailed procedures for drug-induced retinal degeneration and their confirmation (Choi et al., 2023). The remaining three monkeys served as controls. All procedures were performed in compliance with the ARRIVE guidelines. This study was approved by the Institutional Animal Care and Use Committee of Osong Medical Innovation Foundation, Cheongju, Republic of Korea (KBIO-IACUC-2020-054-4).

The retinas were cut into approximately 2 mm × 2 mm patches and mounted on an MEA (60pMEA200/30iR-Ti, Multichannel Systems GmbH, Reutlingen, Germany) with the ganglion cell layer facing down. The retinal patches’ eccentricity for MEA recording was 4∼8 mm from the fovea as shown in our previous publication [Figure 1 of Cha et al. (2023)]. All the patches were placed in an artificial cerebrospinal fluid solution (124 mM NaCl, 10 mM glucose, 1.15 mM KH_2_PO_4_, 25 mM NaHCO_3_, 1.15 mM MgSO_4_, 2.5 mM CaCl_2_, and 5 mM KCl, all purchased from Sigma-Aldrich, St. Louis, MO, United States), bubbled with 95% O_2_ and 5% CO_2_ to maintain a pH of 7.3–7.4 at 25°C under near-infrared (IR) illumination.

### 2.2 *In vitro* recording and stimulation

The MEA contained 64 circular electrodes in an 8 × 8 grid layout with electrode diameters of 30 μm and interelectrode distances of 200 μm. The electrodes were coated with porous titanium nitride and embedded in a perforated polyimide foil that provided sufficient oxygen and nutrient supply to the retina. Recordings of retinal activity were obtained from multielectrodes with a bandwidth ranging from 1 to 3,000 Hz at a gain of 1,200. The data-sampling rate was 25 kHz for each electrode.

**FIGURE 1:**
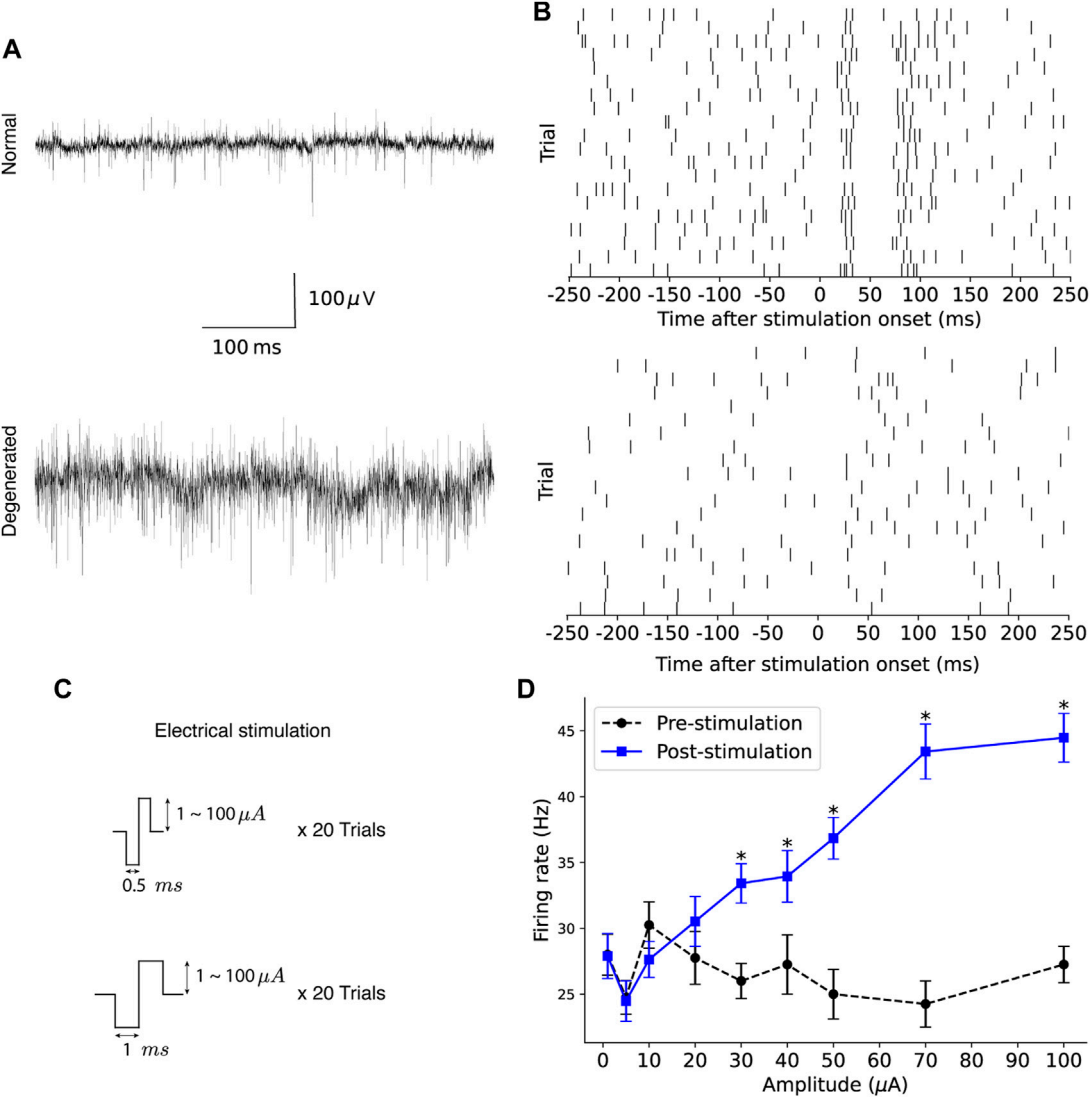
Experimental protocol to assess the modulation efficiency of RGCs. **(A)** Typical raw traces from recordings of spontaneous activity of normal (top) and degenerated (bottom) RGC, respectively, are shown. **(B)** Raster plots of normal (top) and degenerated (bottom) RGC spikes in response to repeated electrical stimulation are shown. **(C)** Electrical stimulation was delivered using symmetric biphasic pulses of varying amplitude and duration. **(D)** An example of pre- and post-stimulation firing rates is shown as a function of stimulation amplitude, where the error bar indicates the standard error of the trial-to-trial mean and the asterisk indicates the statistically significant increase in post-stimulation firing rate compared to pre-stimulation firing rate (*p* < 0.05, one-tailed paired t-test).

After waiting 20 min for the retinal tissue on the MEA to stabilize, spontaneous RGC responses were recorded without electrical stimulation for approximately 30 s. Figure 1A shows typical raw traces from normal (top) and degenerated (bottom) RGC recordings. Electrical stimulation generated by a stimulus generator (STG 1004, Multichannel Systems GmbH, Reutlingen, Germany) was applied to the retinal patch through one electrode, while the other 58 electrodes recorded the RGCs extracellularly. Figure 1B shows the raster plots of the normal RGC (top) and the degenerated RGC (bottom) in response to repeated trials.

Electrical stimulation consisted of symmetrical biphasic pulses of varying amplitudes and durations (Figure 1C). Pulse duration was either 0.5 or 1 ms, and pulse amplitudes of 1, 5, 10, 20, 30, 40, 50, 70, and 100 μA were applied for each duration, resulting in total 18 stimulation conditions. Biphasic current pulses were applied 20 times, once per second (1 Hz), for each stimulation condition.

### 2.3 Data analysis

The spike times of the RGCs were preprocessed as follows: The raw MEA recording trace was high-pass filtered with a cutoff frequency of 100 Hz, and spike sorting was performed on the filtered signal using Offline Sorter™ v4 (Plexon Inc., Dallas, TX, United States) to transform the waveforms containing multiunit activities into multiple single-unit spike trains. The total number of identified cells was 530 normal RGCs and 360 degenerated RGCs. The resulting time stamps of the RGC spikes were analyzed using custom Python codes developed in-house.

Spontaneous firing activity during the 30-second period was quantified as follows: First, the average spontaneous firing rates were calculated. Next, inter-spike intervals (ISIs) were calculated for all spontaneous spikes. The mean and standard deviation of the ISIs were then calculated for ISIs of less than 500 ms.

To focus on the network-mediated RGC responses, we collected the spikes originating from soma of RGCs by electrical stimulation and excluded the axon-stimulating response as follows. First, we used a post-stimulation time window after 10 ms to exclude the stimulus artifact or the direct RGC response occurring within 10 ms after stimulus onset (Sekirnjak et al., 2006; Boinagrov et al., 2014; Ahn et al., 2017). Second, we removed spikes with triphasic waveforms to exclude the recordings from RGC axons far from the stimulating point. This is because spikes recorded from electrodes close to axons in the macaque retina show a typical waveform of triphasic rather than biphasic temporal structure and smaller amplitude spikes than soma stimulation (Madugula et al., 2022).

The modulation efficiency for the electrically evoked spikes was quantified as follows. First, for each electrical stimulation, firing rates were measured for the pre-stimulation (−200∼0 ms) and post-stimulation (10∼200 ms) time windows, denoted by ${FR}_{pre}$ and ${FR}_{post}$ , respectively. Then, reliable modulation of RGCs by electrical stimulation is determined by whether the post-stimulation firing rate is significantly greater than the pre-stimulation firing rate. Specifically, for each stimulation condition, the significance of the increase in firing rate from pre-stimulation to post-stimulation was quantified using the one-tailed paired t-test for 20 trials of ${FR}_{pre}$ and ${FR}_{post}$ with a significance *p*-value of 0.05. For levels of stimulation amplitude ( $n_{amplitude}$ ), the number of such significant increases in firing rate is counted and denoted by $n_{significance}$ . Finally, the ratio of $n_{significance}$ to $n_{amplitude}$ is defined as modulation efficiency ratio (MER) in Eq. 1.

$$MER=\frac{n_{significance}}{n_{amplitude}}.$$ (1)

Figure 1D shows an example of the pre- and post-stimulation firing rates as a function of stimulation amplitude. Black circles with dotted lines indicate the trial-to-trial mean pre-stimulation firing rates. Blue squares with solid lines indicate the trial-to-trial mean post-stimulation firing rates, with the error bar indicating the standard error of the trial-to-trial mean. Both pre- and post-stimulation firing rates showed high trial-to-trial variability. The pre-stimulation firing rates fluctuated for different current amplitudes. In addition, the post-stimulation firing rates were close to or even lower than the corresponding pre-stimulation firing rates when the stimulation amplitude was low. Among nine stimulation amplitude values ( $\left.n_{amplitnineude}=9\right)$ , the post-stimulation firing rate was significantly greater than the pre-stimulation firing rate for the five stimulation amplitudes ( $n_{significance}$ = 5), indicated by asterisks in Figure 1D. Thus, MER was 5/9 for this example.

If there were more than two modes in the given histogram (Figure 3B), the centers of the modes were calculated using the Gaussian mixture model (Parsons, 2020). Specifically, the k-means clustering algorithm (MacQueen, 1967; Jain, 2010) was used to obtain an initial estimate of the centers. The centers were then iteratively updated using the expectation-maximization algorithm (Dempster et al., 1977; Xu and Jordan, 1996) until convergence.

For the analysis of the bimodal histograms of MER shown in Figure 4B, if the given histogram was bimodal, the threshold separating the two modes was determined using the Otsu algorithm (Otsu, 1979) as follows: First, the within-class variance values were calculated for all possible thresholds. For instance, the number of significant increase (numerator of the MER) can take integer values ranging from zero to eight. Therefore, the possible threshold values are 0.5/9, 1.5/9, … , and 8.5/9. Next, the threshold that minimized the within-class variance was chosen as the optimal threshold.

For the analysis of the ISIs shown in Figure 7, histograms of the ISIs were fitted as follows: For normal RGCs, histograms of ISIs were fitted with a gamma distribution, which models the exponential decay and the refractory periods (Dayan and Abbott, 2005). Degenerated RGCs tended to show an additional peak in the ISI histogram at approximately 50 ms, corresponding to spikes synchronized with the oscillatory rhythms in degenerated retinal networks (Goo et al., 2016; Ahn et al., 2022). Therefore, the ISI histogram of the degenerated RGCs was fitted using a mixture of gamma and Gaussian distributions.

The association between the predictors of interest and the MER was evaluated (Table 1). Linear regression was performed using internal and external variables as predictors. The statistical significance of the association between a given predictor and MER was assessed at a significance level of 0.05. The null hypothesis was that there was no association between the predictor and MER (coefficient = 0). If the *p*-value of the coefficient was < 0.05, the null hypothesis was rejected, indicating that changes in the predictor were significantly associated with MER.

**TABLE 1:** Linear regression of MER onto external and internal variables (n.s.: *p* > 0.05, ***: *p* < 0.001, **: *p* < 0.01, *: *p* < 0.05).

Group	Estimated coefficient	
	Normal	Degenerated
Inter-electrode distance	−0.002***	−0.002***
Spontaneous firing rate	−0.0006^n.s.^	−0.0024^n.s.^
Mean of ISI	0.0003^n.s.^	0.0012***
The standard deviation of ISI	0.0004^n.s.^	0.0009*
CV_ISI_	−0.08^n.s.^	−0.28**

ISI, inter-spike interval; CV, coefficient of variation; n.s., no significance.

## 3 Results

### 3.1 Comparison of spontaneous firing properties between normal and degenerated RGCs

**FIGURE 2:**
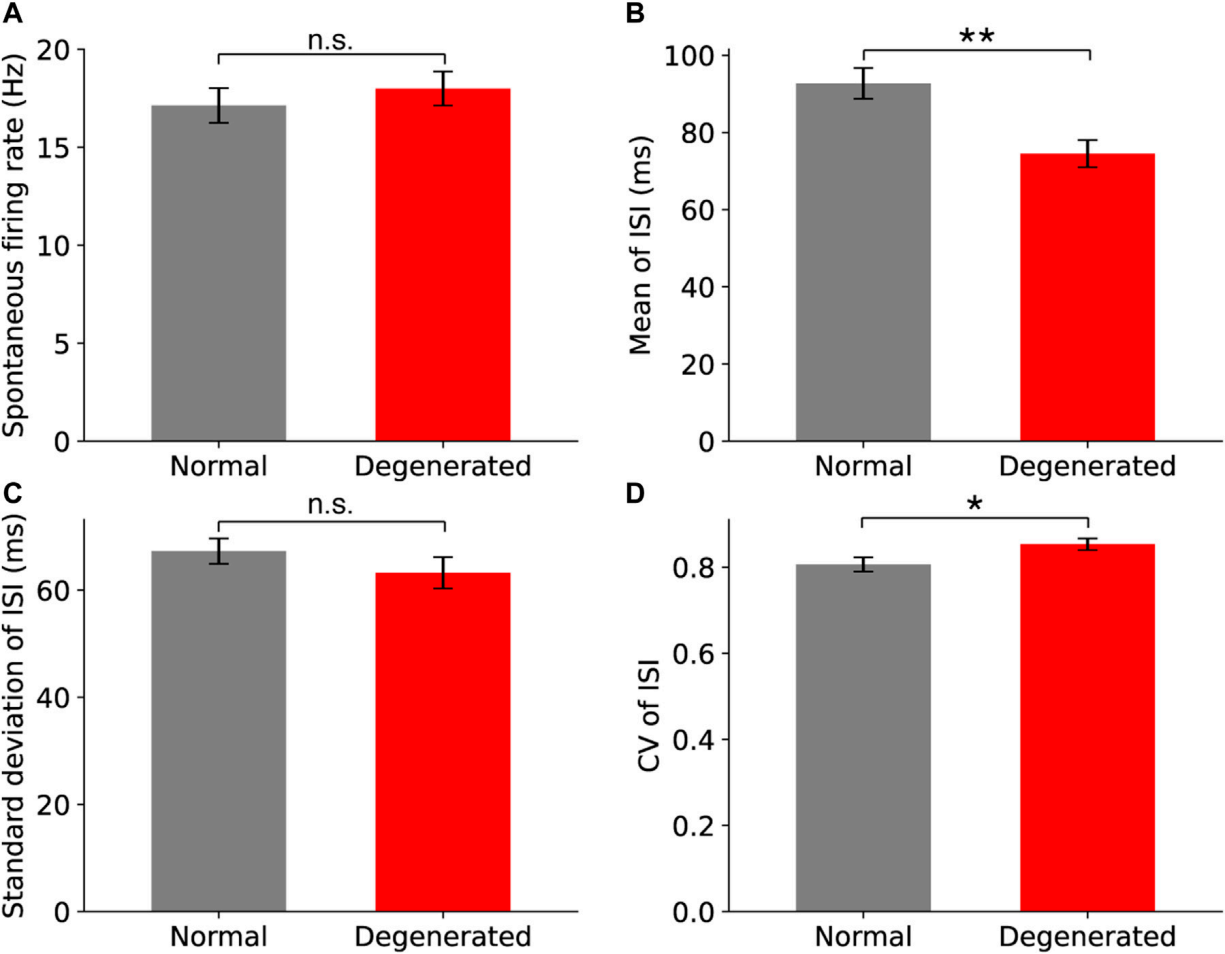
Comparison of spontaneous firing properties between normal (gray) and degenerated (red) RGCs for spontaneous firing rate **(A)**, the mean ISI **(B)**, the standard deviation of ISI **(C)**, and the coefficient of variation (CV) of ISI **(D)**. The error bar represents the standard error of the mean. Statistical significance was assessed using the t-test: asterisks and n.s. represent statistical significance (**: *p* < 0.01, *: *p* < 0.05) and no significance (*p* > 0.05), respectively.

Among the four internal variables of spontaneous firing, the mean and CV of the ISIs were significantly different between normal and degenerated RGCs. The spontaneous firing properties of normal (gray) and degenerated (red) RGCs are compared in Figure 2. Spontaneous firing rates measured for 30 s did not differ between normal and degenerated RGCs (*p* > 0.05, t-test, Figure 2A). In contrast, the mean ISI of the degenerated RGCs was significantly shorter than that of the normal RGCs (**p < 0.01, t-test; Figure 2B). Thus, degenerated RGCs tended to generate more spikes within a shorter time interval than normal RGCs did. The standard deviations (SDs) of ISI did not differ between normal and degenerated RGCs (n.s.: *p* > 0.05, t-test, Figure 2C). The CV of the ISI of the degenerated RGCs was higher than that of the normal RGCs (*p < 0.05, t-test; Figure 2D). This is because CV is the ratio of the SDs to the mean. A lower mean ISI and the same SDs resulted in a higher CV.

**FIGURE 3:**
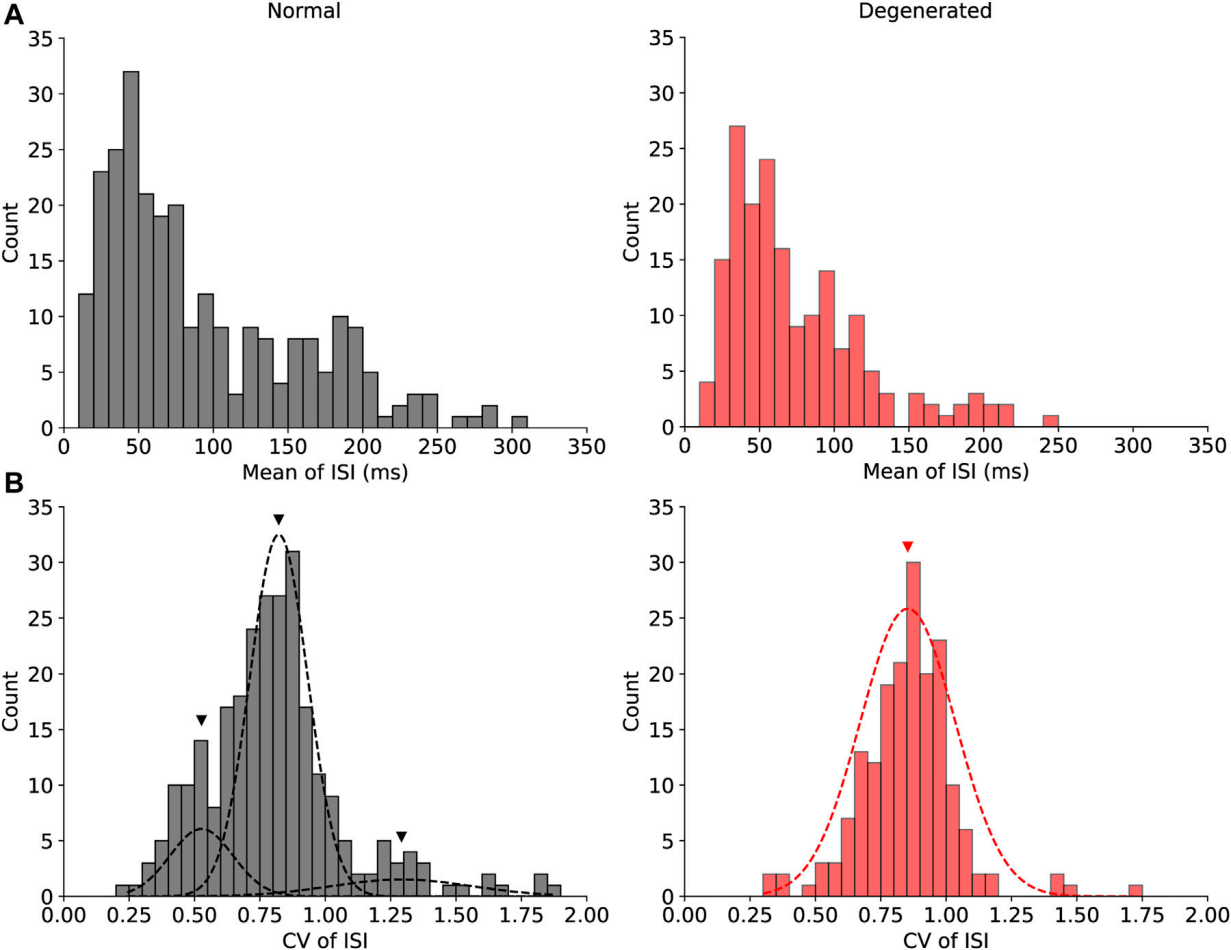
Histograms of the mean ISI **(A)** and the CV of ISI **(B)** show significant differences between the normal (gray) and degenerated (red) RGCs. The distribution of the CV of ISI in normal RGCs [**(B)**, gray] was multimodal, and it was fitted with the Gaussian mixture model with three modes. Black triangles indicate the means of the three modes of normal RGCs (0.53, 0.82, and 1.29). The red triangle indicates the mean of the one mode of degenerated RGCs (0.85).

Figure 3 shows the histograms of the two internal variables that showed significant differences between the normal and degenerated RGCs. Figure 3A shows the histograms of the mean ISI of normal (gray) and degenerated (red) RGCs. First, the normal RGCs (gray) had a wider range of mean ISIs than the degenerated RGCs (red) had. Secondly, degenerated RGCs were more likely to have shorter mean ISIs (<0.15 s) and less likely to have longer mean ISIs (>0.15 s) than normal RGCs were. Consequently, the distributions of mean ISIs were significantly different between normal and degenerated RGCs (*p* < 0.05, Mann-Whitney U test).

Figure 3B shows histograms of the CV of the ISI for normal (gray) and degenerated (red) RGCs. The distributions of the CV of ISI were significantly different between the normal and the degenerated RGCs (*p* < 0.001, Mann-Whitney U test). In particular, the distribution of the CV of the ISI in normal RGCs was multimodal. Figure 3B shows the three modes of the normal RGC distributions fitted with the Gaussian mixture model as black dashed curves, with black triangles indicating the centers of the three modes. The middle mode (mean = 0.82) of the normal RGCs was the most common, followed by the lower (mean = 0.53) and upper (mean = 1.29) modes. In contrast, the CV of the ISI of the degenerated RGCs had a unimodal distribution with a mean of 0.85 and was marked by a red triangle, which was close to the main mode of normal RGCs. Thus, the degenerated RGCs showed less diversity with respect to the CV of the ISI.

### 3.2 Assessment of modulation efficiency of normal vs. degenerated RGCs

**FIGURE 4:**
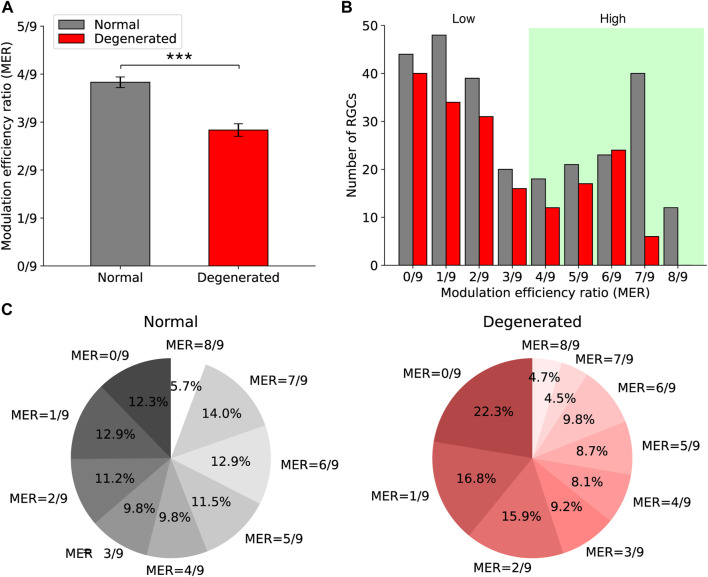
Comparison of modulation efficiency ratios (MER) between normal and degenerated RGCs. **(A)** The bar graph shows the mean MER of normal (gray) and degenerated (red) RGCs. Error bars are standard errors of the mean. The asterisk indicates the statistical significance (***: *p* < 0.001, t-test). **(B)** The histograms of MER of normal (gray) and degenerated (red) RGCs are shown. **(C)** The proportions of MER in normal (left) and degenerated (right) RGCs are shown.

The degenerated RGCs had lower MER than normal RGCs had. Figure 4A shows the MER of normal (gray) and degenerated (red) RGCs, with error bars representing the standard error of the mean (SEM). The mean MER of each group was 3.8/9 (normal) and 2.8/9 (degenerated). The difference between the means was statistically significant (*p* < 0.001, t-test).

Furthermore, the MER of both normal and degenerated RGCs showed a bimodal distribution. Figure 4B shows histograms of the MER of normal (gray) and degenerated (red) RGCs when the stimulation duration was 0.5 ms. The optimal threshold that separates MER values into two classes was 3.5/9 for both normal and degenerated RGCs. Thus, the high MER region is shown in the green region in Figure 4B.

Figure 4C shows the proportions of MER with a stimulation duration of 0.5 ms for each group. Fewer degenerated RGCs had higher MER than the normal group had. For the normal RGCs, 54% had an MER greater than 3.5/9 (Figure 4C, left). For the degenerated RGCs, 36% had an MER greater than 3.5/9 (Figure 4C, right).

### 3.3 Effects of external variables on the modulation efficiency of the electrical stimulation

**FIGURE 5:**
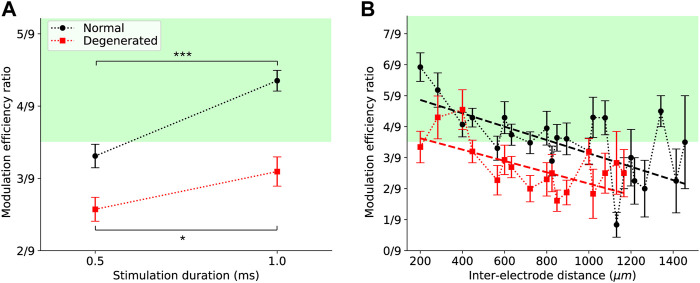
Effects of external variables on the modulation efficiency ratio (MER) of electrical stimulation. **(A)** MER increased for both normal (black) and degenerated (red) RGCs when stimulation duration was increased from 0.5 to 1 ms. Error bars are standard errors of the mean. Asterisks indicate statistical significance (*p* < 0.001 (***) for normal and *p* < 0.05 (*) for degenerated, t-test). **(B)** MER tended to decrease with increasing distance between stimulating and recording electrodes for both normal (black) and degenerated (red) RGCs. Error bars represent standard errors of the mean. Dashed lines are linear regression fits.

Longer stimulation durations increased MER in both normal and degenerated RGCs, although the degree of increase in MER efficiency differed between the groups. Figure 5A shows MER as a function of stimulation duration, with error bars indicating SEM. When stimulation duration was increased from 0.5 to 1 ms, the mean MER of normal RGCs increased from 3.3/9 to 4.4/9 (black circles). This increase was statistically significant (***p < 0.001, t-test), indicating that most RGCs were modulated by a higher charge. T-test was used to assess statistical significance because the stimulation duration had only two values. Similarly, the MER of degenerated RGCs increased from 2.6/9 to 3.1/9 with longer stimulation durations (red squares). This increase was statistically significant (*p < 0.05, t-test), yet less than that of the normal RGCs. Additionally, the average MER of degenerated RGCs remained below 3.5/9.

MER decreased as the distance between the stimulation and recording electrodes increased. Figure 5B shows the MER as a function of the inter-electrode distance, with error bars indicating the SEM. For normal RGCs (black circles), the MER was highest (5.9/9) at the shortest inter-electrode distance (200 μm) and then decreased as the inter-electrode distance increased. The slope of the linear regression fit (black dashed line) was −0.002 μm^−1^ and significantly different from zero (Table 1). For degenerated RGCs (red squares), the highest MER (4.6/9) was achieved when the inter-electrode distance was slightly farther (400 μm). The linear regression fit of the degenerated RGCs (red dashed line) showed a similar slope (−0.002 μm^−1^) to that of the normal RGCs (Table 1).

### 3.4 Effects of internal variables on the modulation efficiency of the electrical stimulation

**FIGURE 6:**
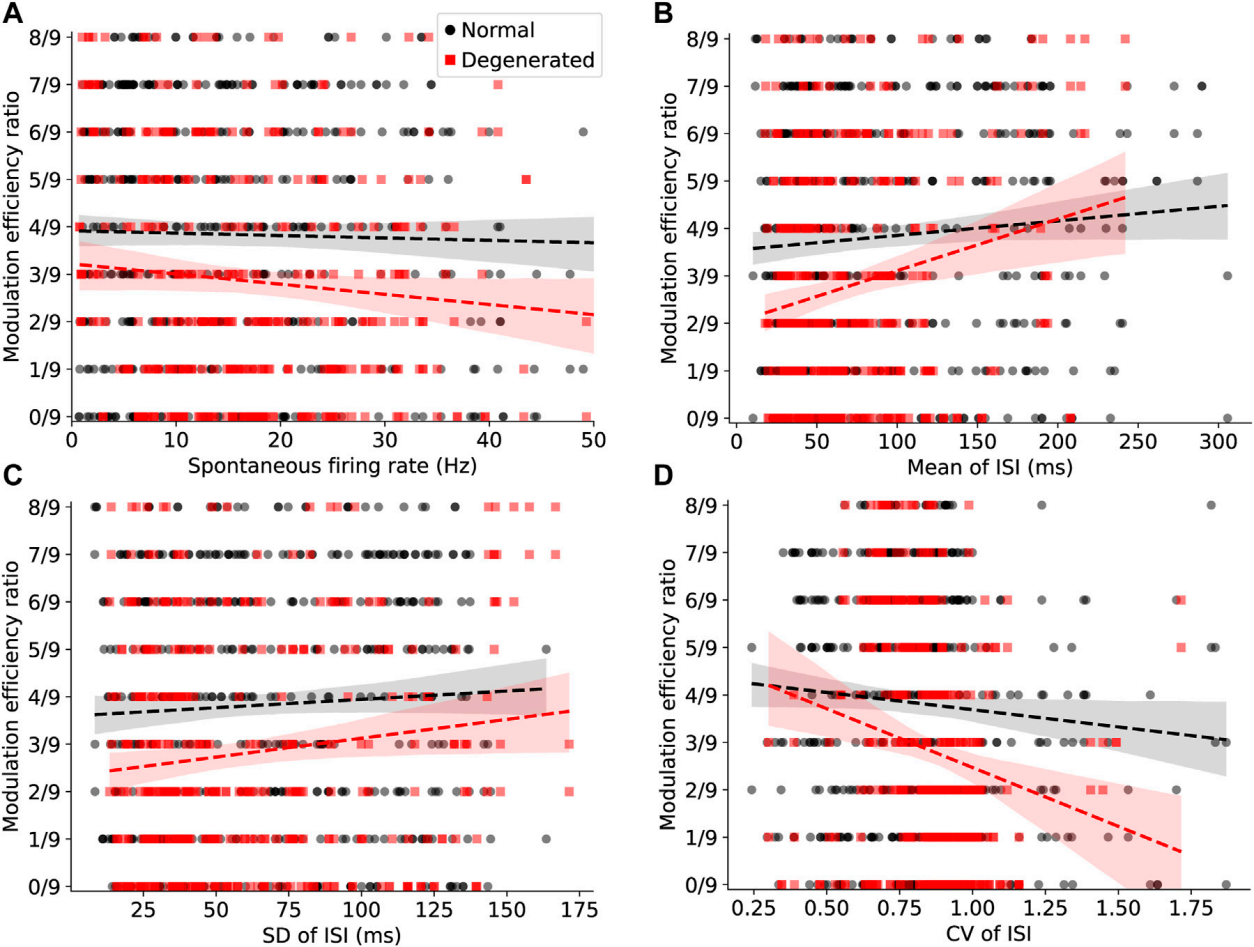
Effects of internal variables on the modulation efficiency of electrical stimulation. Scatter plots of MER and internal variables for normal (black) and degenerated (red) RGCs are shown, with regression lines (dashed) and 95% confidence regions (shaded). **(A)** Spontaneous firing rates of RGCs were not associated with MER for both normal (black) and degenerated (red) RGCs (*p* > 0.05, linear regression). **(B)** The mean of the inter-spike interval (ISI) was positively correlated with MER only in degenerated RGCs (*p* < 0.05, linear regression) but not in normal RGCs (*p* > 0.05, linear regression). **(C)** The standard deviation (SD) of ISI was positively correlated with MER only in degenerated RGCs (*p* < 0.05, linear regression) but not in normal RGCs (*p* > 0.05, linear regression). **(D)** The CV of ISI was negatively correlated with MER only in degenerated RGCs (*p* < 0.05, linear regression) but not in normal RGCs (*p* > 0.05, linear regression).

While the internal variables were not associated with MER in normal RGCs, the properties of inter-spike intervals were associated with MER in degenerated RGCs. Figure 6 shows scatterplots of the MER and internal variables for the normal (black) and the degenerated (red) RGCs, with regression lines (dashed) and 95% confidence regions (shaded). The estimated coefficients of the regression fit are listed in Table 1. The relationship between the MER and each internal variable is as follows.

Spontaneous firing rates of RGCs were not associated with MER in either the normal or the degenerated RGCs. The regression lines (dashed lines) in Figure 6A show a subtle negative correlation with MER in both normal (black) and degenerated (red) RGCs. However, this correlation is not statistically significant, as demonstrated by the *p*-values of the estimated coefficients in Table 1.

The mean inter-spike interval was positively correlated with MER in degenerated RGCs; however, it was not in normal RGCs. In Figure 6B, the regression line (black dashed line) of normal RGCs shows a slightly positive correlation with MER. However, this positive relationship was not statistically significant in normal RGCs (n.s., Table 1). In contrast, the regression line (red dashed line) of degenerated RGCs showed a strong positive correlation with MER, which was statistically significant (***p < 0.001; Table 1). The estimated coefficient of the mean ISI was 0.0012 (Table 1).

Similarly, the SD of the ISI was positively correlated with MER only in degenerated RGCs but not in normal RGCs. In Figure 6C, the regression line (black dashed line) of normal RGCs shows a slightly positive correlation with MER. However, this positive relationship was not statistically significant in normal RGCs (n.s., Table 1). In contrast, the regression line (red dashed line) of degenerated RGCs showed a stronger positive correlation with MER, which was statistically significant (*p < 0.05, Table 1).

The CV of the ISI was negatively correlated with MER only in degenerated RGCs but not in normal RGCs. In Figure 6D, the regression line (black dashed line) of normal RGCs shows a slightly negative correlation with MER. However, this negative relationship was not statistically significant in normal RGCs (n.s., Table 1). In contrast, the regression line (red dashed line) of the degenerated RGCs showed a stronger negative correlation with MER, which was statistically significant (**p < 0.01; Table 1).

**FIGURE 7:**
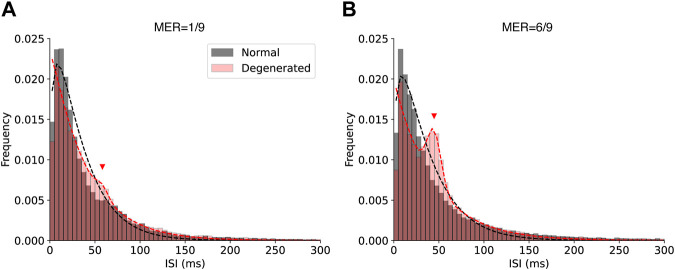
Histograms of the ISI of normal (black) and degenerated (red) RGCs with low **(A)** and high **(B)** modulation efficiency. Black dashed curves represent gamma distribution fits for the normal RGCs. Red dashed curves represent fits using a mixture of gamma and normal distributions for the degenerated RGCs. Red triangles indicate the additional peak fit by the normal distribution.

Figure 7 shows the histograms of the ISI of RGCs with low (A) and high (B) modulation efficiencies. The ISI of normal RGCs showed a single peak at a very small value and decayed rapidly at larger values at both low and high MER (Figures 7A, B). This shape fits with the gamma distribution (black dashed curves). In contrast, the ISI histogram of degenerated RGCs showed an extra bump at approximately 50 ms (red triangles in Figure 7), which was more prominent at a higher MER (Figure 7B). The gamma and normal distribution mixtures fit the multimodal histograms (dashed red curves).

## 4 Discussion

Previous studies on the effective activation of RGCs using electrical stimulation have significantly advanced our understanding of retinal prostheses. These investigations have explored various aspects of electrical stimulation, including electrode design, stimulation parameters, and the intricacies of RGC responses to electrical pulses (Jensen et al., 2003; Jensen et al., 2005; Fried et al., 2006; Jensen and Rizzo, 2008; Sekirnjak et al., 2008; Jensen and Rizzo, 2009; Goo et al., 2011a; Habib et al., 2013; Jepson et al., 2013; Abramian et al., 2014; Ahn et al., 2015; Weitz et al., 2015; Ahn et al., 2022; Ahn et al., 2023; Cha et al., 2023; Gogliettino et al., 2023; Seo et al., 2024). The results of these studies provide critical insights into optimizing the efficacy of retinal prostheses to achieve precise and reliable activation of RGCs.

However, a simple comparison of different experiments cannot show how well electrical stimulation evokes RGC spikes. This study examines the physiological characteristics that may determine the efficiency of electrical stimulation and suggests that both external and internal variables play important roles in the MER.

### 4.1 MER is a reliable criterion for quantifying modulation efficiency

MER is a robust metric to quantify the efficiency of electrical stimulation in both normal and degenerated RGCs. MER is based on a statistically significant increase in post-stimulation firing rates compared with pre-stimulation firing rates measured in repeated trials. Therefore, MER is robust to the inherent variability in neural responses and experimental artifacts. More importantly, MER quantifies the electrical stimulation efficiency with a normalized value between zero and one for each RGC, whereas previous thresholding methods provided information about the threshold *per se* without distinguishing subtle differences in electrical stimulation efficiency. In general, the stimulation threshold is defined as the stimulation setting that corresponds to a predefined (typically 50%) probability of eliciting an RGC spike (Cai et al., 2013; Boinagrov et al., 2014; Lorach et al., 2015). Another group defined the stimulation threshold as the current setting producing a > 90% probability of an RGC spike (Sekirnjak et al., 2006). In our previous study (Goo et al., 2011b; Cha et al., 2023), the efficiency of modulation was indirectly measured by interpolating the current amplitude of the stimulation, eliciting an RGC spike with a 50% probability. This approach is inherently unfavorable for a degenerated retina when quantifying modulation efficiency because a degenerated retina has fewer evoked RGC spikes per pulse than normal RGCs have. Consequently, the degenerated RGCs showed higher stimulation thresholds than the normal RGCs, regardless of the species (Cha et al., 2022; Ahn et al., 2023; Cha et al., 2023). The stimulation threshold provides information on the minimum intensity of electrical stimulation, which differs significantly between normal and degenerated RGCs.

In order to compare modulation efficiency ratios of different experiments, the stimulation amplitudes should be fixed. The MER would change for different choices of current amplitude values. This dependence of the modulation efficiency score on the specific choice of amplitude values could be reduced by using widely used current amplitude values. In our experiment, the safety limit determines the maximal current amplitude, resulting in a typical range of 0 to 100 uA. This study used nine current amplitude levels spread over 0∼100 uA. For the lower current amplitude range (1∼10 uA), more minute intervals of amplitudes were chosen (1, 5, and 10 uA). While for the higher current amplitude range (50∼100 uA), more sparse intervals of amplitudes were chosen (50, 70, and 100 uA). This selection was based on the need to cover a wide range of amplitudes while ensuring safety and accuracy in our measurements.

The bimodal distribution of MER in both the normal and the degenerated RGCs (Figure 4B) supports the use of MER as a criterion for discriminating between high- and low-efficiency electrical modulation. The degenerated RGCs produced fewer spikes than the normal RGCs did in response to electrical stimulation at the same intensity, resulting in a lower average MER (Figure 4A). Nevertheless, the MER of the degenerated RGCs showed a bimodal distribution and a clear distinction between the high- and low-efficiency groups, demonstrating the feasibility of MER for analyzing individual RGCs. Therefore, MER is a reliable quantification criterion for both normal and degenerated RGCs.

We examined the external and internal variables related to MER to determine the conditions associated with higher modulation efficiency. The relationship between MER and external variables showed a similar trend for both normal and degenerated RGCs, but with different efficacies. A longer stimulation duration and shorter distance resulted in a higher electrical charge delivered to the RGCs, and as a result, a higher MER (Figure 5; Table 1). In contrast, internal variables were differentially associated with normal and degenerated RGCs. In the normal retina, internal variables did not affect MER (Figure 6; Table 1). Three internal variables (mean ISI, SD of ISI, and CV of ISI) were significantly associated with MER in the degenerated retinas. Among the three internal variables, the mean ISI is the most important. RGCs with relatively longer mean ISIs showed less hyperactivity. Therefore, they may serve as suitable targets for electrical stimulation.

### 4.2 Spontaneous firing of RGC spikes between normal and degenerated retinas

Spontaneous hyperactivity in RGCs is a well-known characteristic of degenerate retinas (Goo et al., 2011a; Sekirnjak et al., 2011; Stasheff et al., 2011; Soto et al., 2012; Yee et al., 2012; Margolis et al., 2014). Hyperactive firing of spontaneous RGC spikes has been observed in a genetic model and an n-methyl-n-nitrosourea (MNU)-induced degeneration mouse model (Tao et al., 2015). NHPs are ideal animal models because of their anatomical and physiological similarities to humans. However, a genetic model of retinal degeneration in primates is lacking. Iatrogenic NHP retinal degeneration (RD) models have been developed using chemical- and laser-induced methods (Shirai et al., 2016; Ou et al., 2018; Dhakal et al., 2020; McGregor et al., 2020; Choi et al., 2023).

Our previous NHP RD model study reported that spontaneous RGC spike firing in monkeys with MNU-induced RD was significantly higher than that in normal monkeys (Ahn et al., 2022).

However, in the present study, spontaneous RGC spike firing in normal and RD primates was not significantly different (Figure 2A).

There are several possible explanations for this disparity in results. One of the most important possibilities is the selection of RGCs. In our previous study, we counted the RGC in the pool when it showed a typical RGC spike form, as shown in Figure 1A of Ahn et al. (2022). We excluded the RGC in a normal retina if it fires spikes extraordinarily high. However, in this study, we counted all the RGCs even if they fired too many spontaneous spikes because we observed spontaneous hyperactive firing from patch to patch, not scarcely in normal retinas. In addition, we attempted to exclude any experimenter-based bias in cell pooling. Second, more animals and retinal patches were used in this study (animal number: normal = 3, RD = 3; retinal patch number: normal = 5, RD = 4) than in the previous study (animal number: normal = 3, RD = 2; retinal patch number: normal = 3, RD = 2). Additional animals and retinal patches can provide more objective findings. Third, the MNU-induced morphological changes in the outer nuclear layer, as confirmed by OCT findings [Figure 2 in Cha et al. (2023)] looked similar. However, degeneration-induced network changes in secondary neurons (such as bipolar and horizontal cells) were not the same in each RD case, inevitably resulting in different RGC spike firing patterns.

### 4.3 Difference in the mean ISI between normal and degenerated retinas

In this study, the responses of RGCs were summed, and their subtypes were not differentiated. Visual stimulus-driven classification should be performed to differentiate the RGC subtypes. Because we did not apply a visual stimulus for this experiment, we pooled all RGCs identified through spike waveform sorting without differentiating the RGC types.

Our ISI profile of the normal retinas showed a more heterogeneous distribution than that of the RD retinas (Figure 3). As proposed in our previous publication using an *rd10* mouse (Cha et al., 2022), if the ON response is preserved for longer than the OFF response with MNU-induced RD, more ON cells could remain. This ON dominance leads to a more homogeneous distribution in the RD ISI profile. Previous primate research has focused only on the direct response of RGC regarding retinal prosthesis (Sekirnjak et al., 2008; Jepson et al., 2013; Gogliettino et al., 2023). Because our RGC spikes were derived from indirect network-mediated responses rather than direct responses, there may be a difference in RGC responses to electrical stimulation. During RD, there is a significant rewiring of synaptic connections in the inner retina (Marc et al., 2003). Gap junctions are essential for the generation of abnormal rhythmic bursts and oscillations in *rd10* RGCs (Toychiev et al., 2013; Menzler et al., 2014; Ivanova et al., 2016). Gap junction coupling between AII amacrine cells and ON-cone BCs is known to be involved in oscillation generation (Trenholm et al., 2012; Yee et al., 2012; Choi et al., 2014). These complex changes could affect the mean ISI in primates with RD.

We could better understand cell-type-specific responses if we linked the RGC subtype identified with the visual stimulus and each RGC subtype’s response to electrical stimulation. This issue will be addressed in future studies.

### 4.4 Effect of external variables on MER between normal and degenerated retinas

This study investigated the effects of two external variables, stimulation pulse duration and inter-electrode distance, on MER.

A more prolonged stimulation pulse significantly increased the MER in normal and RD retinas (Figure 5A). This can be easily explained by the two-fold increase in the charge applied to the retina. However, MER still falls under the MER value of 3.5/9 in the RD retina. These findings are compatible with our previous report [Figure 5 of Cha et al. (2023)], in that in RD retina, the evoked spikes of RGC barely cross the threshold value (0.5). They were consistently below the levels of the normal retina across all current amplitudes tested.

The MER in both the normal and RD retinas showed an inverse linear relationship with increased inter-electrode distance. The decrease in modulation efficiency with increasing interelectrode distance is consistent with the intuition that distant RGCs receive less electrical charge and, therefore, have a lower MER (Figure 5B).

An inversely linear curve along the inter-electrode distance in the RD retina seems to contradict the normalized RGC response with the distance between stimulation and recording electrodes in RD macaques [Figure 4C of Ahn et al. (2022)], which shows a widespread distribution of electrically evoked RGC populations. In this study, the MER was derived from the RGC response with nine different amplitudes tested and not from each current amplitude, as shown in Figure 4C of Ahn et al. (2022). If we integrate all the current amplitudes (10, 30, and 50 μA), the previous Figure 4C of RD macaque could be similarly incorporated with our current Figure 5.

### 4.5 Effect of internal variables on MER between normal and degenerated retinas

This study examined the effects of four internal variables, spontaneous firing rates, mean of ISI, SD of ISI, and CV of ISI, on MER. Among the four internal variables, the statistic of ISI was significantly associated with the MER of degenerated RGCs. Specifically, degenerated RGCs with shorter ISIs tended to have lower MER (Figure 6B).

Degenerated RGCs showed a bump of around 50 ms in the ISI histogram (Figure 7). A possible physiological mechanism that generated this bump is the oscillatory membrane potential of degenerated RGCs. The origin of this oscillation is the electrical coupling between ON bipolar cells and AII amacrine cells in the degenerated retina (Borowska et al., 2011; Choi et al., 2014; Trenholm and Awatramani, 2015), which is supported by empirical evidence that gap junction blockers abolish such rhythmic activity of degenerated RGCs in mice. The same phenomenon has been observed in drug-induced retinal degeneration in rabbits (Ahn et al., 2019) and macaques (Ahn et al., 2022). Specifically, in Figure 1D of Ahn et al. (2022), the power spectral density of the local field potential of degenerated macaque RGCs showed a strong peak around 20 Hz, which corresponds to the bump around 50 ms in the inter-spike interval in our experiment.

A novel contribution of our study is that the degenerated RGCs with lower modulation efficiency tend to have shorter ISIs than 50 ms (∼20 Hz). The synchronous firing around 20 Hz is the hallmark of degenerated RGCs. Our results show that there are degenerated RGCs that have prominent oscillatory spikes but still have higher modulation efficiency (Figure 7B). This suggests that more careful studies should be performed to dissociate the effects of oscillatory spikes driven by the electrical coupling and hyperactivity on a shorter time scale. Understanding the physiological mechanisms of the low modulation efficiency of hyperactive RGCs is an important future work. The modulation efficiency of the generated RGCs could be improved by suppressing the oscillatory drive from AII amacrine cells or by reducing the hyperactivity of the RGC itself.

### 4.6 Feasibility of MER for *in vitro* MEA recording and clinical setting

We selected the stimulation channel in the middle of the MEA, considering the voltage gradient produced around the recording channel. Based on our results, we propose the following strategy for selecting the optimal stimulation channel for *in vitro* MEA recordings: First, record spontaneous RGC activity from all available channels of the MEA without electrical stimulation for a sufficient period (e.g., 30 s). Second, perform spike-sorting to obtain spontaneous spikes from individual RGCs. Third, calculate the mean ISIs of RGCs over a short time interval (e.g., 0.15 s) and identify RGCs with large mean ISI values. Fourth, identify candidate stimulation channels near RGCs with large mean ISIs. Finally, apply electrical stimulation to each candidate channel.

In this study, only one MEA channel was used for stimulation. In such scenarios, selecting the channel closest to the RGCs with the largest mean ISI is a promising strategy. Electrical stimulation can be simultaneously applied to multiple channels (Ryu et al., 2017). The spatial arrangement of candidate channels and the average ISIs of RGCs around the channels should be considered for multichannel stimulation to maximize modulation efficiency. Moreover, the results of this study may also have implications for more advanced devices. For instance, the CMOS MEA, with a high spatial resolution, is used to investigate the functional properties of the retinal network (Stutzki et al., 2016; Cojocaru et al., 2022). Considering the ISI of RGCs and their spatial arrangement in such a setup could improve electrical stimulation efficiency.

We propose that the MER can be one method for calibrating retinal prostheses. Several uncertainties and challenges arise when an MEA device is implanted into a living retina. For example, an immune response may lead to inflammation and gliosis near the implanted device, and the displacement or corrosion of the MEA device can change the quality of the signal. Our results showed that the response of RGCs to electrical stimulation can be predicted based on the MER.

Therefore, the MER can be used to optimize the selection of stimulation channels for *in vitro* MEA experiments and practical calibration methods to achieve higher efficiency when testing retinal prostheses.

### 4.7 Conclusion

Quantifying the effective activation of RGCs by electrical stimulation is a bottleneck in the study of retinal prostheses under diverse experimental conditions. Therefore, we proposed a robust indicator, the MER, to assess the modulation efficiency of RGCs. To our knowledge, this is the first study to directly compare the responses of normal and degenerated primate RGCs to electrical stimulation, based on a common criterion. We selected two external variables (stimulation duration and inter-electrode distance) and four internal variables (spontaneous firing rate, the mean ISI, SD of ISI, and CV of ISI) as predictors of MER in normal and degenerated primate RGCs. Two external variables significantly affected the MER in normal and degenerated RGC (*p* < 0.05). Three internal variables, such as the mean ISI, SD of ISI, and CV of ISI, significantly affected MER (*p* < 0.05) in degenerated RGC but not in normal RGC. The most important variable affecting MER was the mean ISI. A shorter ISI indicates hyperactive firing in the degenerated retina, which prevents electrical stimulation from evoking more RGCs. We believe that this difference in spontaneous firing in the RD results in a lower MER than that in the normal retina. Selective recruitment of a less hyperactive RGC group in RD with electrical stimulation could significantly improve the efficacy of the retinal prosthesis.

In future research, exploring additional modulation-efficiency predictors will be helpful. A broader range of variables should be considered in future studies, including cell type, morphology, and genetic markers and their relationships with MER. This comprehensive approach may provide a more detailed understanding of RGC modulation. In addition, *in vivo* studies are required to further understand the modulation efficiency and improve the efficacy of retinal prostheses. Such studies are essential for assessing the complex dynamics of the living retina and the long-term viability of retinal implants. We expect MER to be a common feature when comparing RGC responses in both *in vitro* and *in vivo* experiments.

## Data Availability

The raw data supporting the conclusions of this article will be made available by the authors, without undue reservation.
